# The Internet of Things Term Feature Extraction Method and Its Application in English-Chinese Translation

**DOI:** 10.1155/2022/2134627

**Published:** 2022-04-15

**Authors:** Lili Jian

**Affiliations:** Department of Foreign Languages, Wuhan College, Wuhan 430212, China

## Abstract

From the emergence of the concept of the Internet of Things to today, China has always been very concerned about the development of the Internet of Things technology. The research and application of Internet of Things technology has become an important measure for the country to promote supply-side reform. The Internet of Things technology is the core innovation content of the country and an important measure to realize the smart city. This paper analyzes the cutting-edge research results in the field of Internet of Things abroad. This paper focuses on the application of IoT technology in English terminology research. This paper specifically analyzes the feature extraction methods of Internet of Things terms and studies the application content of Internet of Things technology in English-Chinese translation. We specifically conduct translation research on the content in “The Internet of Things: Enabling Technologies, Platforms, and Use Cases,” focusing on the translation of scientific and technological terms. As a kind of technical English terminology, IoT English terminology has the characteristics of strong professionalism and rigorous semantics. This article specifically translates the original part of the practice report and classifies the terms that appear in it, including IoT English terms for which canonical translations have been made and IoT English terms that do not have standardized translations. The research will further carry out the investigation and analysis work. The research is to identify the terms that have standardized translation, especially the detailed research of the terms of the industry field. This paper selects standard translations and summarizes the translation methods of terms from three aspects: abbreviations, compound words, and semi-technical words. The research results provide guidance for terminology translation. This article translates terms that do not yet have a canonical translation. Based on terminology characteristics and existing terminology translation methods, this paper proposes a variety of translation methods such as literal translation, decomposing and combining, and non-translation. The study described in detail with examples. The research results of this paper are expected to provide some reference for translators engaged in the translation of scientific and technological documents.

## 1. Introduction

The material selected for this translation is “Internet of Things: Enabling Technologies, Platforms, and Use Cases,” which is a cutting-edge monograph in the field of Internet of Things, published by CRC Press in 2017. The publishing house is a publishing institution in the field of professional textbooks and reference books. The publications of this press have high academic value. With the development of science and technology, the Internet has profoundly affected all aspects of people's lives [1–3]. Especially in the 21st century, the rapid development of technology has promoted the wide application of network technology. Therefore, the Internet of Things was born, and it has become one of the hottest topics at present. However, the theoretical system of the Internet of Things is not sound enough. There are relatively many theoretical and practical research studies on Internet of Things management, which has always been the focus of domestic and foreign academic and business circles. Experts in different fields think differently about IoT. This book is a comprehensive introduction to the Internet of Things technology, covering the basic concepts, development history, and technical security of the Internet of Things. The readers of this book can be both professionals in the field of Internet of Things and general readers who are interested in Internet of Things technology. This study further translates the book into Chinese, which is of great significance for popularizing the knowledge of the Internet of Things. It has a good reference value for promoting research in the related fields of the Internet of Things [4–7].

IT experts said that in the future, the technology industry will develop science and technology having no loopholes which can spontaneously integrate [8–11]. On this basis, scholars create a technology cluster that can proactively meet professional requirements. Therefore, we need to organically integrate our own thinking. In the later stage of the development of the concept of the Internet of Things, scholars gradually realized the interconnection between the various elements of the Internet of Things. Because of electronic devices, we will have real-time deep access to the world's information assets. On the other hand, we will have permanent backups of our brains in massive digital storage. A huge amount of research has been put together into the seemingly miraculous cutting-edge technologies that will beneficially connect our nervous systems to computers [12–15]. Disruptive and transformative technologies will emerge with the intelligent synchronization of vast amounts of information and communication technologies. The use of revolutionary applications and social networks is becoming more frequent. The digital knowledge society is constantly growing. Personal data are being consolidated, and automatic identification tags with preferences are widely used [16–19].

These labels are continually drawn, merged, and made available to all. The application of labels satisfies new human desires. The everyday tools and products we use are constantly evolving. These products can be converted into smart products by connecting to ultra-small computers. For example, when we are interacting with other products or humans nearby, our coffee cups, dinner plates, tablets, and clothes will be empowered with intelligent behavior. In the end, all tangible, valuable things will be transformed into intelligent, sentient digital products [20]. Together, these elements make up the Internet of Things for decades to come. As a result, future generations will experience and realize a fully and reliable digital and technology-driven life. It includes other tech-enabled lives, tech-sponsored lives, and tech-prosperous lives. The impact of IT on our lives has grown over time.

Information technology has provided a beneficial improvement to the efficiency of enterprise management and complex business processing. With the continuous introduction of information technology, it has begun to pay attention to the needs of people. As listed below, there are several noteworthy improvements in the IT world. So, the human-centered shift is right. That is, it is not just business services and applications. A conceptual analysis of human-centered services is also required. This kind of service can perfectly realize the people-centered service content and then maintain the people-centered service goal. Some of the best algorithms, patterns, platforms, processes, and products are refactored. This article expands into the human-centered IT field in a smooth manner. There are a number of highly anticipated trends in IT. This paper continues to deepen in this area. Therefore, in that era, our daily life will become the basic building block, specifically related to audio or video systems, cameras, and information and networking equipment. These include consumer and household electronics and other electronic devices. Content includes digitized walls, floors, windows, doors, ceilings, and other physical objects and products.

**Figure 1:**
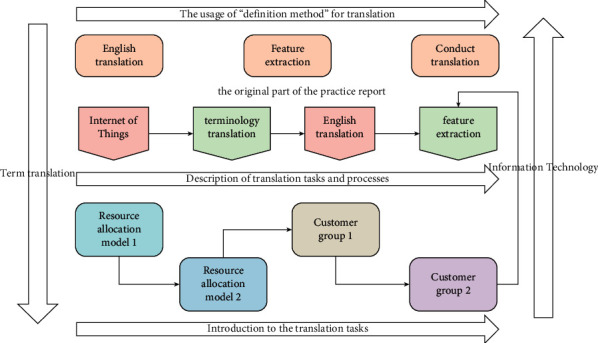
The research logical structure of this paper.

Computers, communicators, sensors, and robots are constantly developed. These devices can actively provide human beings with various daily needs. Devices can continuously motivate or support researchers in their decision making. At the same time, scholars have also discovered the importance of the impact of the external environment of the Internet of Things on it. In order to meet our daily manual labor, humanized robots will be widely used. In conclusion, IoT technologies synchronized with cloud infrastructure will enable human-centric intelligent environments. Specifically, situational awareness is a key driver for various businesses and IT systems to differentiate their operations and outputs. Based on the translation of the book “Internet of Things,” this practice report extracts and organizes the terms appearing in the book. This paper further summarizes the main methods of terminology translation. While introducing the foreign frontier research on the Internet of Things, it also provides some reference for the terminology translation work of scientific and technological texts. The research logical structure of this paper is shown in Figure 1.

## 2. Description of Translation Tasks and Processes

This article introduces the translation task, further describes the translated text, introduces the translation tools, and sorts out the translation process. Specifically, this practice report is introduced from four aspects.

### 2.1. Introduction to the Translation Tasks

**Figure 2:**
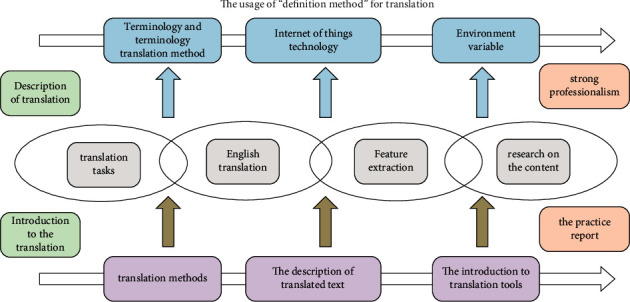
The relationship diagram of the translation tasks and processes.

Terminology is an important part of scientific texts. Standardized and strict use of terminology is the basic premise of scientific research information exchange. Accurate terminology translation is the key to the conversion of scientific and technological information between languages. Terminology translation quality largely determines the translation quality of scientific and technical texts. Since the advent of translation, people have recognized the importance of terminology translation. Chinese scholar Yan Fu proposed in the preface of the “New Dictionary of General Encyclopedia” that terminology is the essence of the national language. The noun concept here mainly refers to proper nouns. The Internet of Things is an emerging technology field, and professional texts in this field have typical scientific and technological stylistic characteristics. In addition, there are also related research studies where terminology translation plays an important role in translation work. Terminology translation is not only the starting point of translation work but also the purpose of translation work, so it must be taken seriously. Model analysis results are shown in Figure 2.

### 2.2. The Description of Translated Text

The book “Internet of Things” is divided into two parts. The first part focuses on the development history, main concepts, and key technologies and protocols of the Internet of Things. The second part of the book focuses on the integration of IoT with various industries, for example, the application of the Internet of Things in the airport, the application in the health care system, etc. Finally, the book discusses security threats and protection techniques related to the Internet of Things.

The title of the first part of this article is “Demystifying the Internet of Things Paradigm,” mainly to explain the strategic significance of the Internet of Things and the changes that have taken place in the field of Internet of Things and further provide the trend judgment of the future development of the Internet of Things. The second part, titled “Using Wireless Technologies to Enable the IoT Ecosystem,” discusses the core wireless technologies that exist in the IoT space. Then, the focus is on low-power wide area network technology. These technologies are widely used in the IoT ecosystem to enable interconnectivity between devices and applications. It provides help for the comprehensive analysis of belonging. Three types of application examples related to the IoT ecosystem are specifically introduced, including industrial cases, consumer cases, and management cases of IoT. The vocabulary used in the Internet of Things is highly specialized. In the consumer IoT case section, the article focuses on three applications in smart home, smart building, and smart education. The article discusses the practical forms of smart cities in the management case section.

### 2.3. The Introduction to Translation Tools

In order to improve the speed and quality of translation, this translation practice relies on tools and materials such as machine-assisted translation software, dictionary reference books, and professional literature in the field.

**Figure 3:**
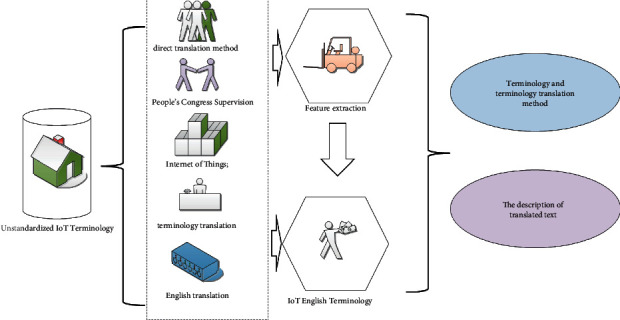
Schematic diagram of the dynamic pricing of consumption and durable goods revenue management.

1. *Machine-Assisted Translation Tools*. SDL Trados machine-assisted translation software was used to assist translation software. This article uses this tool to import the original text to be translated, convert the document format, and translate in English sentence units. During the translation process, this article stores the translated text and terminology separately and stores them in the translation memory. The information in the database provides reference for subsequent translations and supports the consistency and standardization of translations before and after the article. Specifically, the use of machine-assisted translation tools can better extract terms in the text.
2. *Dictionary Tool*. Dictionary tools mainly include paper dictionaries and online dictionaries. The paper dictionary mainly refers to the “English-Chinese Dictionary of Science and Technology,” which is an authoritative material published in 2009. Common words mainly rely on online translation tools to search for definitions and translations. Online translation software Youdao Dictionary is a representative of this field. This translation software includes a number of dictionaries, such as Collins, Oxford, and Webster. Through this software, one query can get the explanation of a word in different dictionaries. This query method, on the one hand, can save the user's query time and, on the other hand, is conducive to selecting the most appropriate word meaning. Professional terms are mainly searched using professional dictionaries and professional websites. As an official terminology query tool, terminology online software is highly authoritative. This software can be used to aid in the search for a term that has a canonical translation.
3. *Professional Field Literature*. This article collects and organizes relevant professional literature in the field of Internet of Things through China National Knowledge Infrastructure (CNKI). These documents provide support for better understanding and translating the professional content. Professional vocabulary and terminology in the field of IoT tend to be complex. Long length, difficult memory, and inconvenient reading and writing are major characteristics of technical terms. Some parts of the professional literature collected include the Internet of Things White Paper (2018). This document is used to understand the development trend of IoT and learn the language of scientific and technological texts. The second book is “Introduction to the Internet of Things,” which is a university textbook published in 2011 and a popular science book on the Internet of Things. This book covers tags, sensors, smart devices, and more. This document is very helpful for understanding the English original text of “Internet of Things.” The book “Smart City: Internet of Things Architecture and Application” was published in 2014. This book not only introduces the related technologies of the Internet of Things but also introduces the application fields of the Internet of Things in detail. The book focuses on smart home, smart medical care, smart transportation, and smart grid. The book introduces IoT applications. The interrelationships between model elements are shown in Figure 3.

Through this translation practice, the author realizes the importance of terminology translation. At the same time, this paper also has a new understanding of the pretranslation preparation work. The article finds its own shortcomings, as well as the limitations of the proposed translation method. The research of this paper has accumulated experience for future translation work.

## 3. The Terminology and IoT English Terminology

This section first introduces terms and their translation methods and then analyzes the characteristics of IoT English terminology. Finally, based on the existing terminology translation methods and IoT terminology characteristics, this paper proposes a translation method for IoT English terminology.

### 3.1. Terminology and Terminology Translation Method

Terminology refers to the specialized terms of specialized disciplines and scientificity which are the primary characteristics of terminology. Secondly, the terminology also has the characteristics of being systematic in the field. Under the influence of this factor, many of the Internet of Things English terms are expressed in acronyms. Terminology translation should not only show the characteristics of IoT terminology but also involve the issue of cross-cultural correspondence in this article. In his 2019 paper, Li Changchun argued that treating terminology translation should be like engaging in scientific research. The translation of terms should be guided by scientific methods, through repeated investigation and verification, to find an equivalent or almost completely equivalent translation method. Terminology translation work should not trust the translation given by encyclopedia entries. Terminology translation is very important both from a linguistic point of view and from a professional point of view. Terminology translation has attracted many researchers to carry out related research work. Existing studies have proposed many valuable terminology translation methods. In 2012, Zheng Shupu divided terminology translation into “regulated terms” and “unregulated terms.” A regulated term means that the term already has a definite translation name and cannot be taken for granted when translating. Normative terms must be translated in accordance with the normative documents published by the relevant agencies.

**Figure 4:**
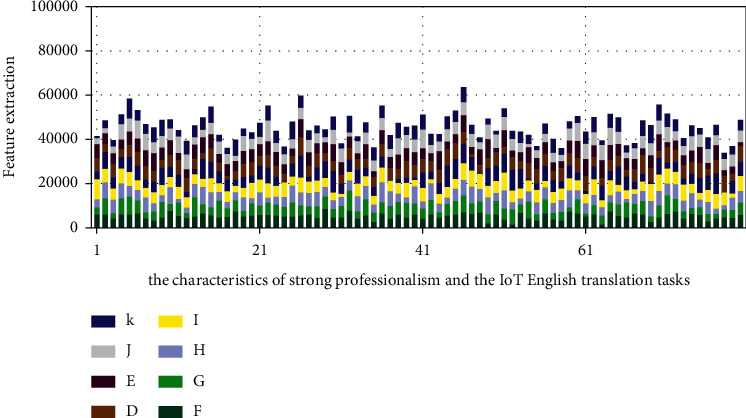
The method of combining disassembly and translation.

In July 2018, the Ministry of Civil Affairs issued the “Announcement on the Translation of Standard Chinese Characters for Place Names.” The purpose of this announcement is to standardize the translation of place names. By doing normalized translations, the randomness of terminology translation is avoided, ensuring accurate expression and transmission of terminology information. It refers to the fact that the current term translation is not officially confirmed. Translators need to give appropriate translation results according to the connotation of terms. The Internet of Things is a branch of the computer category, and a large part of the Internet of Things terminology is consistent with the computer terminology. Zheng Shupu summed up some feasible methods in a lot of practice. There are four methods including “no translation method,” “trial translation method,” “definition method,” and “concatenation method.” Model analysis results are shown in Figure 4.

**Figure 5:**
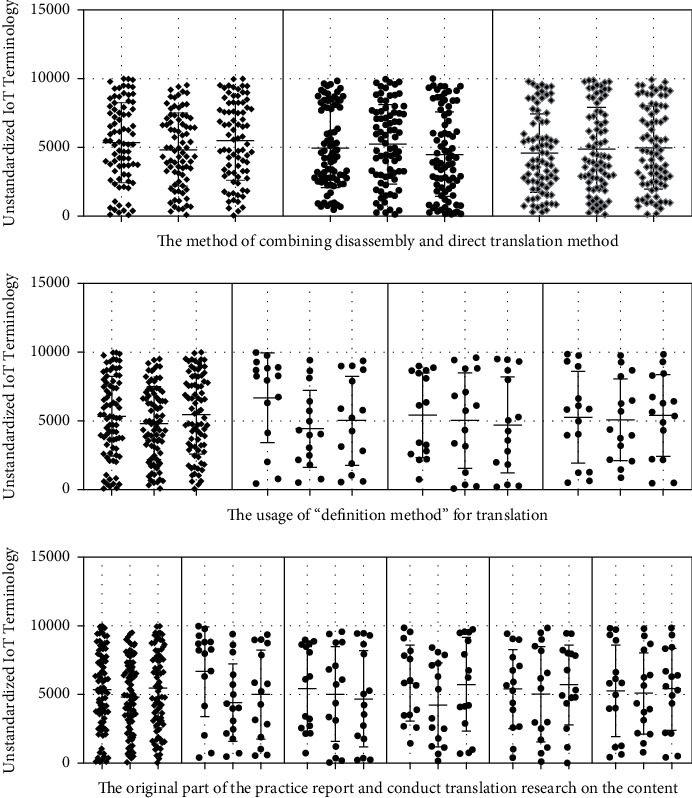
The method of using the original English text directly.

**Figure 6:**
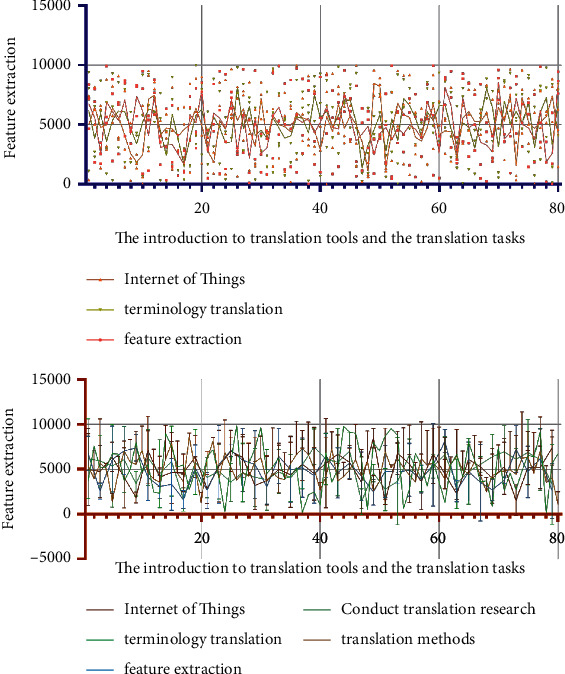
The method for combining translations with multiple methods.

1. “Untranslated method” refers to copying the original terminology. This method is not a translation of inaction, but a last resort. There are many precedents in the existing translation work. Especially in the field of science and technology, the translation is carried out by direct quotation of the original text. For example, there is a type of integrated development software, the English name is Eclipse, and the corresponding Chinese term is Eclipse. The use of this English name in the computer field is deeply ingrained. If these words are translated literally, it will cause readers to misunderstand. The Eclipse environment is easier to understand by users and the developers. Eclipse software is also easily accepted in the computer field. In addition, just like SDL Trados software, the software's Chinese name is Tados. Some IoT terms are very similar in composition to computer terminology. We often use the software that can express semantics in the translation field. Model analysis results are shown in Figure 5.
2. The “trial translation method” is to mark the original text after the translation has been carried out. The purpose of this is to inform the reader that the current translation is for reference only. The original text of the annotated can facilitate the reader's understanding and encourage the academic community to come up with better translations. In this paper, this method is most directly reflected in the translation of book titles (The Internet of Things: Enabling Technologies, Platforms, and Use Cases). At present, the mode of translation is not clarified in the official translation (primarily unregulated terms). A similar term is expressed in the book General Practice and Community Health Terminology (First Edition). The main meaning of this translation is the factor that promotes a certain behavioral maneuver or desire, so that the desire can be realized. The technologies and resources are necessary to achieve a certain behavior. In Pedagogy Terminology (first edition), a similar term “enabling objective” is directly translated as “enabling objective.” This definition is the stage goal that the learner must achieve to reach the teaching end goal. However, these stage goals have not yet reached the corresponding secondary teaching goals. In different disciplines, the translation and expression effects of the same English word are different. Model analysis results are shown in Figure 6.

Since the author's research on the Internet of Things is still relatively shallow, it is currently impossible to determine the translation of enabling in the Internet of Things. This article translates “Enabling Technologies” as “technology.” This article further translates it as “Technologies.” Obviously, there is a lack of meaning in this translation method. Therefore, this paper marks the original text after the translation. The advantage is to facilitate readers to inquire the book. At this point, additional definitions or explanations may be used herein to further clarify the terms. Readers can obtain more complete information inquiries. Avoid translation bias that affects access to information.

### 3.2. The Usage of “Definition Method” for Translation

**Figure 7:**
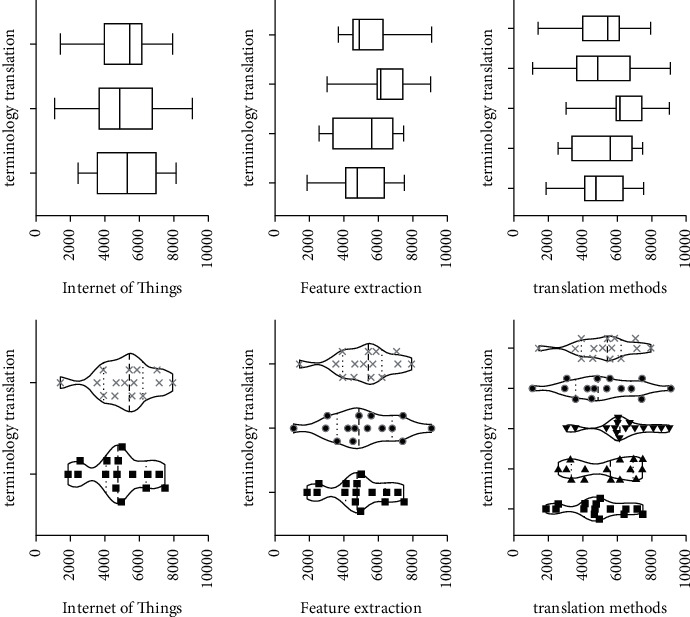
Conducting translation research on the content.

Zheng Shupu clarified the concept of translation in 2012. Specifically, the translation content is expressed in an appropriate manner. For example, by adding brackets or adding annotations, the translation or the original text of the definition of the term is provided to the reader. There are many benefits to doing so. When the translator does not know enough about the relevant profession, he cannot understand the connotation of the term from the surface text of the term translation. Translators give readers a better understanding of the meaning of terms by giving clear definitions. Model analysis results are shown in Figure 7.

**Figure 8:**
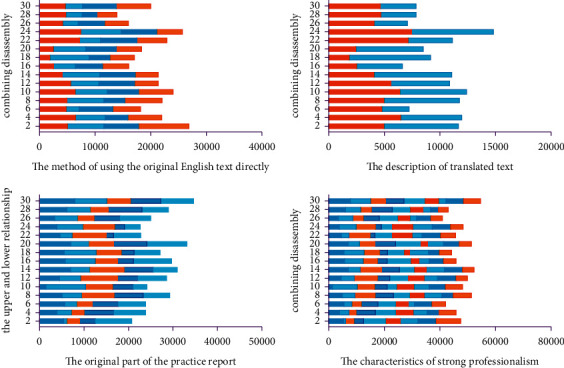
The introduction to translation tools and the translation tasks.

The methodology systematically brings together terms and their definitions in an appendix. Such a method is referred to as the “concatenation method.” Because some terms have a superordinate-subordinate relationship between many concepts, this article links the terms with the upper and lower relationship, which is beneficial to show the connection between the terms. Before the concept of terms was made completely clear, this article linked the word alphabetically. Taking this approach helps to look up terminology. This practice report contains a terminology appendix. When readers are reading, if they need to check the terminology appendix in advance, they can quickly grasp the keywords. In addition, some scholars have proposed translation methods such as literal translation, free translation, demolition translation, and restoration in the “Translation of Aerospace English Terminology” published in 2011. Combining the examples of aerospace English terminology, this paper proposes a translation method based on literal translation. Further flexibility is to select other translation methods and other different viewpoints. In 2015, Zhang Yanping and Wang Guilian advocated the use of free translation, transliteration, and simultaneous translation of unregulated terms for translation. Model analysis results are shown in Figure 8.

**Figure 9:**
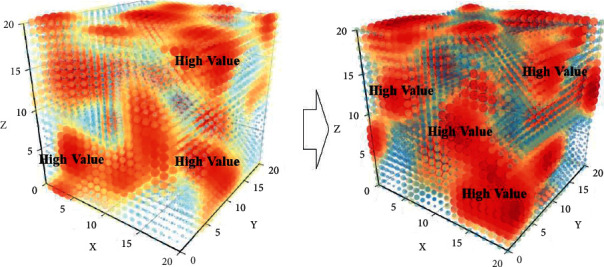
The characteristics of strong professionalism and the IoT English translation tasks.

In general, although the terminology translation strategy proposed in this paper is not perfect, it can complete this translation practice well. For the Internet of Things English terminology that does not have standardized translations encountered in practice, the research results in this paper can provide directions for the translation of Internet of Things terms and then promote the dissemination of the science and technology. Model analysis results are shown in Figure 9.

### 3.3. The Terminology Features of IoT English

**Figure 10:**
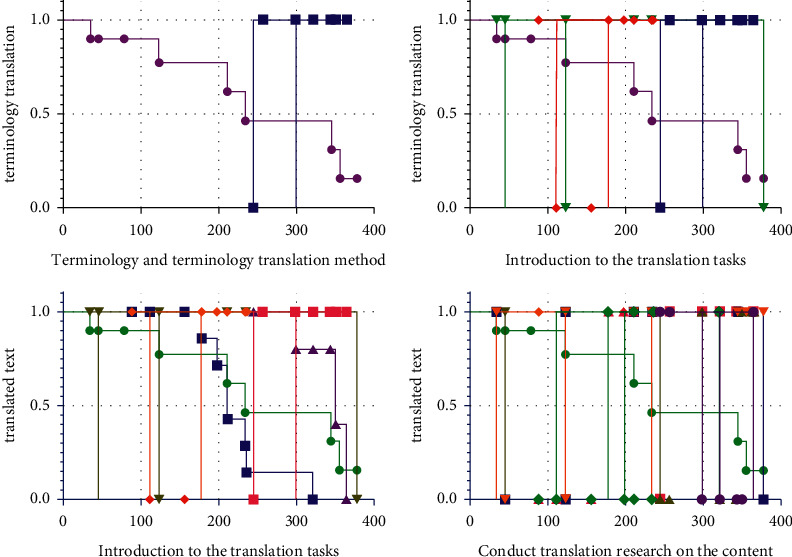
The English translation and feature extraction in the field of IoT.

The Internet of Things is an emerging technological field, and professional texts in this field have typical scientific and technological stylistic characteristics. The vocabulary used in the Internet of Things is specialized. This professional vocabulary is more complex than the previous word organization. In addition to specialized vocabulary, there is also some interdisciplinary technical vocabulary appearing more frequently. Therefore, there are many acronyms in IoT English terminology, for example, IoT, 5G, NFC, QoE, etc. However, acronyms can sometimes create a multiword situation. For example, NFC could potentially be translated as near-field communication and as non-condensed material. Non-condensed material is not a recognized term within the context of the Internet of Things. The term is more often used in the production of juice. On other occasions, the term is translated as the National Football Association. This issue requires translators to pay special attention. Acronyms can be correctly identified by this method, so that a correct translation can be given. Such semi-technical terms are ambiguous and have very flexible collocations. Therefore, under the same major, there may be different translations of words. For example, speck is often translated as “dot” and “smudge” in everyday situations. However, the term is translated as “particles” in IoT English. At the same time, there is also a technology called intelligent particle technology, which is different from the translation of this term in other fields. Bus is no longer the meaning of “bus” in the English terminology of the Internet of Things, but it should be translated as “bus.” Model analysis results are shown in Figure 10.

**Figure 11:**
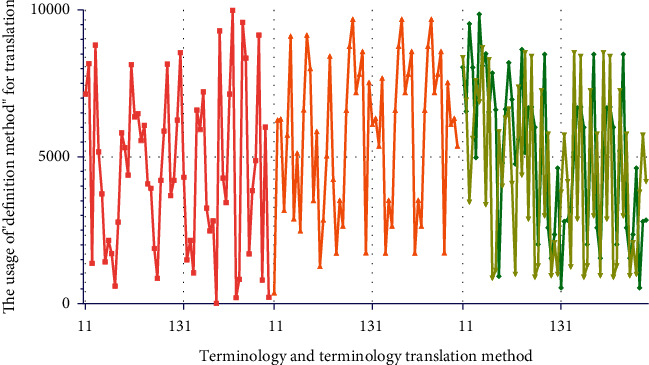
Analysis results of terminology and terminology translation method.

This translation practice report first analyzes the importance of terminology in translation. Secondly, this paper discusses the translation methods of different scholars on different terms. On this basis, this article takes “The Internet of Things: Enabling Technologies, Platforms, and Use Cases” as an example to translate the English terminology of the Internet of Things. This paper further starts from the terms of the existing standardized translation and analyzes the terms that do not have standardized translation. The research further clarifies the overall strategy for terminology translation. At present, the existing terms on standardized translation are mainly divided into three categories: abbreviations, compound words, and semi-technical words. Terms under different categories have different translation methods. The translation methods summarized in this paper can guide the terminology that does not have standardized translation. This practice report examines terms that are not standardized in the original translation. This paper argues that the accuracy and transparency of translations are the primary principles of research. According to the research content, this paper further proposes the literal translation method, the dismantling and combination method, the non-translation method, and the combination of various translation methods. Model analysis results are shown in Figure 11.

### 3.4. The Translation Methods of IoT English Terms

Bill Gates first proposed the concept of the Internet of Things, and the technology used in this concept originated from Western countries. Therefore, the technical terms about the Internet of Things are mostly foreign words. On the one hand, such words need to be translated. Compared with traditional Chinese words, these foreign words have some differences in the translation process. In particular, there are some terms that sound a bit odd, such as “cloud computing.” The English word is cloud computing, and the Chinese translation of the word is a literal translation from English. The concept of this type of noun is relatively more abstract, and it is difficult to understand its inner meaning through direct translation of the text. However, from the reader's point of view, when the reader reads the technology starting with “cloud,” he does not need to understand the complex technology and structural system behind it. Therefore, malfeasance needs to regard “cloud” as a common name. For an in-depth understanding of the cloud concepts, we can read more. The approach we are now familiar with goes further. For us general readers, we may not write the Java language, but we all know Java languages can implement many applications. The Internet of Things is a branch of the computer category, and a large part of the Internet of Things terminology is consistent with the computer terminology. There are also some terms in the Internet of Things that are very similar to the terminology of computer science. Therefore, we can learn from the translation of computer terminology and translate some IoT English terms by analogy. The translation results obtained in this way are relatively more reliable and accurate. This method of translation allows terminology translation to be consistent within disciplines, meeting the basic requirements for terminology usage and translation. Because IoT English belongs to technical English, the characteristics of technical English should be reflected in the translation of IoT English terms, for example, the professionalism of words and the rigor of logic. According to whether the terms have standardized translations, this paper divides IoT English terms into two categories, namely, IoT English terms with standardized translations and IoT English terms without standardized translations, and applies corresponding translation methods according to the term categories.

## 4. Conclusion

### 4.1. Summary of Article Translation Methods

Terminology concepts are core concepts in professional fields and bridges for the exchange of ideas and knowledge. The accuracy of this article's translation of terms may affect the speed at which technology spreads. The English terminology of the Internet of Things includes information technology, communication technology, low power consumption technology, radio frequency identification technology, and so on. Therefore, this paper sorts out the related technologies of translation of Internet of Things. On the one hand, terminology awareness is the prerequisite for scholars to translate terminology, and terminology competence is the guarantee of translation quality. The starting point of the translation practice in this paper is the pretranslation preparation. This article conducts an in-depth analysis by collecting high-quality parallel texts and authoritative terminology. It further clarified that translation tools are an indispensable step in carrying out translation work. After determining the translation materials and the writing direction of the thesis, the author consulted a lot of Internet of Things professional and terminology translation materials, for example, “Introduction to the Internet of Things” and terminology online sites. Specifically, thanks to sufficient pretranslation preparations, the author was able successfully complete this practice report.

In the existing normative translations, IoT English terms are divided into three categories, namely, acronyms, compound words, and semi-technical words. For such terms, their content has been identified in IoT-related translations. It can be commonly followed and used within the industry. The focus of the examination of the translation of such terms is not the accuracy of the translation. In the relatively normative translation, this paper further summarizes the translation method, providing reference for terms that have not been standardized in existing translations. For example, when translating abbreviations, the overall judgment is made according to the popularity of the abbreviation in the country. On the one hand, the full name of the translation of the abbreviation can be used, or the abbreviation can be used directly. Due to the different word formation methods of Chinese and English compound words, the translation of compound words can be divided into word-to-word literal translation and flashback translation. Targeted judgments are made for the Internet of Things English terms that do not have standardized translations. For example, analyze the names of new technologies, new compound words, acronyms, etc. When experts are translating, the comprehensive consideration will be more complicated. Although the term translated by the author is not authoritative at this time, the author's reserve of IoT expertise is relatively rich. At present, the academic community is exploring the translation method of normative terminology. Comprehensively analyze whether the translation method of unregulated terms in academic circles is reasonable. This paper adopts a comprehensive translation method to ensure that the translation of IoT English terms is still accurate and readable when there is no standardized translation.

### 4.2. Problems and Deficiencies in the Translation of This Article

A professional book cannot reflect the characteristics of all terms and the translation methods of terms. In this translation practice, the author only translates some chapters (Chapter 1, Chapter 2, and Chapter 11) of the book “Internet of Things” and extracts terms from them for analysis and summary. The textual materials used in the translation of this article are not rich enough, and the terms involved are not comprehensive enough. This leads to imperfect terminology translation strategies on the other hand. In the research of this paper, there are few examples that can be used in the actual selection of cases. Secondly, with the rapid development of science and technology today, this article involves many cutting-edge platforms and technologies. However, the author is not very clear about the connotation of some terms. Some translations have ambiguities in translation. Therefore, the author consulted many relevant reference texts. In addition, this paper also seeks help from some classmates and teachers and strives to ensure the standardization, science, and rigor of the translation. In addition, there are some charts in the original text, which can systematically and hierarchically display the research content. These diagrams are of great help in describing the technical work process but are not translated in this translation practice. In this way, the hierarchy of different technologies cannot be reflected, which in turn affects the connection between terms and the integrity of the original text.

## Data Availability

The data used to support the findings of this study are available from the corresponding author upon request.
